# Advanced practice nursing role development in primary health care in Brazil and Germany: comparative perspective

**DOI:** 10.1590/0034-7167-2025-0672

**Published:** 2026-10-16

**Authors:** Daria Andrea Bula, Kerstin Hämel, Elyne Montenegro Engstrom, Lígia Giovanella, Vanessa Cappellesso Horevicz Felix, Rosa Maria Rodrigues, Maria Lúcia Frizon Rizzotto, Marcus Heumann, Daniela Rosalba Luisi, Maike Zwergel, Beatriz Rosana Gonçalves de Oliveira Toso

**Affiliations:** I Bielefeld University. Bielefeld, North Westphalia, Germany; II University of Vienna. Vienna, Austria; III Fundação Oswaldo Cruz. Rio de Janeiro, Rio de Janeiro, Brazil; IV Universidade Estadual do Oeste do Paraná. Cascavel, Paraná, Brazil

**Keywords:** Advanced Practice Nursing, Nurse Practitioners, Primary Health Care, Nurse’s Role, Nurse’s Scope of Practice., Enfermería de Práctica Avanzada, Enfermeras Practicantes, Atención Primaria de Salud, Rol de la Enfermera, Enfermera.

## Abstract

**Objective:**

To compare the advanced practice nursing development in primary health care in Brazil and Germany focusing on institutional, historical, and educational factors.

**Methods:**

We conducted a qualitative comparative study combining the analysis of policy documents, literature and secondary data on health systems.

**Results:**

In Brazil, long-standing academic nursing education, established responsibilities, and ongoing debates support advanced practice nursing development, although the absence of a regulatory framework remains a barrier. In Germany, its development reflects a vocational-based nursing tradition and a relatively recent shift toward academization, with few advanced practice nursing master’s study programs. A physician-centered primary care model and insufficient legal regulations constrain implementation, despite growing political momentum.

**Conclusions:**

Advanced practice nursing development is country - and context-specific, meaning there is no one-size-fits-all model; rather, implementation should align with institutional structures, educational pathways, and professional role developments. Effective implementation requires context-sensitive strategies across multiple levels of the health system.

## RESUMEN

**Objetivo:**

Comparar el desarrollo de la Enfermería de Práctica Avanzada (EPA) en la Atención Primaria de Salud en Brasil y Alemania, centrándose en factores institucionales, históricos y educativos.

**Métodos:**

Estudio cualitativo comparativo que combina el análisis de documentos, literatura y datos secundarios sobre sistemas de salud.

**Resultados:**

En Brasil, la tradición en la formación académica de enfermería, las responsabilidades establecidas y los debates respaldan el desarrollo de la EPA, aunque la ausencia de un marco regulatorio sigue siendo un obstáculo. En Alemania, su desarrollo refleja una tradición basada en la formación técnico-profesional y un giro reciente hacia la academicización, con pocos programas de maestría en EPA. Un modelo de atención primaria centrado en el médico y regulaciones insuficientes limitan su implementación, pese al creciente impulso político.

**Conclusiones:**

El desarrollo de EPA es específico de cada contexto y requiere alineación con estructuras institucionales, formación y roles profesionales en sistemas de salud.

## INTRODUCTION

Given the rising prevalence of chronic diseases, demographic aging, and increasing workforce pressures, many countries have intensified efforts to strengthen the role of nurses in primary health care (PHC). Growing evidence shows that nurses contribute significantly to high-quality, patient-centered and community-centered PHC, particularly when they serve as advanced roles such as Advanced Practice Nurses (APNs)^(1)^.

APNs are typically educated at the master’s level and combine advanced clinical competencies with autonomous decision-making and care coordination skills^(2)^. Their role has been adopted in numerous countries and is increasingly seen as a key strategy for addressing complex health needs and increasing PHC capacity^(1)^. Beyond advanced clinical practice, APNs also assume responsibilities in education, leadership, research and service development. However, the integration of nurses into PHC differs markedly across health systems, reflecting differences in institutional, historical, and regulatory contexts^(2)^.

For example, in Brazil, nurses play a key role in PHC since the Family Health Strategy (FHS) established a multiprofessional PHC model. In contrast, in Germany, PHC is provided predominantly by general practitioners (GPs) in private practices, where nurses play no role or only a minor role^(3)^. Consequently, Brazil and Germany represent two fundamentally different PHC delivery models-one that is multiprofessional and nurse-inclusive and another that is physician-led with restricted nursing involvement. However, in both countries, initiatives are currently underway that have the goal of establishing the role of APNs within PHC.

In this paper, we explore and compare “how APN development in PHC is shaped in Brazil and Germany”, and we examine how institutional, historical, and educational factors influence the prospects for APN implementation. To address this research question, we compare the two countries’ nursing histories, educational trajectories, and recent initiatives to introduce APN roles into PHC.

## OBJECTIVE

To compare the advanced practice nursing development in primary health care in Brazil and Germany focusing on institutional, historical, and educational factors.

## METHODS

### Ethical issues

Ethics committee approval is not required for this type of study. Standards for Reporting Qualitative Research (SRQR) were followed by the researchers, when applicable. Although elements of the COREQ were considered to enhance clarity in reporting qualitative procedures, the study design-based exclusively on document analysis-does not fully align with COREQ domains, which are primarily oriented toward interviewand focus group-based research.

### Type of study and theoretical framework

The study was carried out as part of the research project “Strengthening Advanced Practice Nursing and Collaboration in Primary Health Care (APN-PHC).” The aim of the project was to identify the strengths, opportunities, and obstacles of expanding nursing roles and to assess how APN could contribute to advancing PHC in Brazil and Germany. A qualitative comparative design was adopted, which is suitable for examining similarities and differences across distinct national contexts and for generating in-depth, context-sensitive interpretations of complex social and professional phenomena. This approach is particularly suited to understanding how structural, historical, and organizational factors shape professional practices across settings, allowing both within-case and cross-case analysis^(4)^.

We conducted comparative analysis combining a document search and data from (inter)national statistical sources (e.g., the Organization for Economic Co-operation and Development (OECD), the World Health Organization (WHO), and national statistics). The literature and documents included national policy papers, legal frameworks, academic publications, and reports from professional bodies in Brazil and Germany. Developing comparative studies in the field of public policy analysis could be considered recent, dating back to the 1960s/1970s, although it has been a common practice in political science since its beginning^(4)^.

Square 1 provides an overview of the demographic data, health and population indicators, and health care personnel of both countries. Examining these data from a comparative perspective allows us to place different contextual conditions side by side and to learn from the respective experiences of both health systems. In doing so, we draw on a comparative approach widely used across public policy and health systems research. As highlighted in the Bulletin of the World Health Organization, international comparisons of health system characteristics can provide valuable evidence and inform health policy^(5)^.

### Summary of Key Country Characteristics

Differences in demographic, economic, and health-related indicators were detected between Brazil and Germany. Brazil’s population is significantly larger (211,998,573 million vs. 83,510,950 million) and more territorially dispersed, resulting in a much lower population density compared with that of Germany (see Chart 1). Many Brazilian regions-especially in the Amazon and rural areas-are difficult to access, further complicating infrastructure and health care delivery^(6)^. In contrast, Germany has substantially higher levels of economic development, as reflected in its gross domestic product (GDP) per capita (55,800.2 USD vs. 10,280.3 USD), higher life expectancy (81 vs. 76 years), and lower child mortality (3.2 vs. 12.5 per 1,000 live births). Additionally, Germany has a higher density of nursing personnel (12.25 vs. 5.3 nurses per 1,000 inhabitants).

**Chart 1:** Demographic, economic and health indicators in Brazil and Germany. 2025

Indicators	Brazil	Germany
Population	211,998,573 million (2024)	83,510,950 million (2024)
Population growth rate	0.4% (2024)	-0.5 % (2024)
Territorial area	8.509.379,576 km^2^ (2024)	357,596 km^2^ (2022)
Demographic density	25 habitants/km^2^ (2023)	240 habitants/km^2^ (2023)
Gross Domestic Product (GPD)	10,280.3 US dollars (2024)	55,800.2 US dollars (2024)
Life expectancy	76 (2023)	81 (2023)
Fertility rate	1.62 (2023)	1.39 (2023)
Child mortality rate per 1,000 born alive babies	12.5 (2023)	3.2 (2023)
Nursing/1,000 habitants	5.3 (2023)	12.25 (2023)
Nursing salary	4,750 (gross) Real/month^*^	4,329 (gross) Euro/month

Source:^(7-12)^

* Approved by Law number 14.434/22^(13)^.

### Methodological procedures and data analysis

To facilitate comparisons between Brazil and Germany, for data collection, we developed a matrix (Chart 2) that outlines key dimensions of PHC and nursing-covering PHC conception, organization, and services, as well as nursing history, education, and APN development. These were the same themes for the inductive analyses, according to Meny and Thoenig^(4)^.

**Chart 2:** Comparative analysis matrix for health systems, primary health care and advanced-practice nursing.2025

Characteristics of Primary Health Care (PHC)		
**Conception of PHC** What is the concept? What is the role of PHC in the health system?	**Model of care** What is the predominant organization of PHC? What types of services are available in PHC? Which professionals are working in PHC?	**Network integration** What is the role of PHC in the network of health services?
**Characteristics of Nursing**		
**Nursing history** What are the milestones in nursing history?	**Nursing education** How was nursing education developed and what is the current status of nursing education?	**Advanced Practice Nursing (APN)** To what extent has APN been established? What are the main developments, including the most recent ones?

### Study setting and cases

The study focused on two national contexts with distinct health systems and nursing education models, selected based on their relevance to the research objective and their contrasting approaches to APN. This comparative design allows the exploration of how historical trajectories, institutional arrangements, and policy frameworks influence professional autonomy and scope of practice.

### Data sources and document selection

Data consisted exclusively of documentary sources, including national health policies, regulatory frameworks, curricular guidelines, professional standards, and institutional reports related to nursing education and practice. Documents were selected using purposive and criterion-based strategies, prioritizing relevance, credibility, and contemporaneity. Inclusion criteria comprised official publications, peer-reviewed articles, and documents issued by recognized national and international organizations. The selection process sought to ensure sufficient depth and diversity of sources to support robust comparative analysis.

### Data analysis

Data analysis followed a structured, concept-driven approach. An analytical framework was developed a priori to ensure a comprehensive and context-sensitive examination of the research question. Core domains-characteristics of PHC, nursing history and nursing education-guided the data organization. Relevant literature and documents were iteratively assigned to these domains and, where needed, discussed within the binational team to overcome language barriers and ensure accurate interpretation. This approach enabled withinand cross-case comparisons and the identification of convergences, divergences, and explanatory patterns across contexts ^(4,5)^.

## RESULTS

### The Brazilian Case

#### Characteristics of Primary Health Care

In Brazil, PHC is embedded in the Unified Health System (Sistema Único de Saúde - SUS), a universal, tax-funded system established by the 1988 Federal Constitution. SUS guarantees access to comprehensive care and is structured around principles of decentralization, regionalization, and social participation, with municipalities responsible for organizing and delivering PHC services^(14)^. In addition to PHC, SUS also covers specialized and hospital care, emergency services, and high-cost procedures. Within SUS, municipalities oversee PHC services provided in public facilities. Since 1994, a community-oriented PHC model, the Family Health Strategy (FHS), has complemented traditional basic health care units. The FHS addresses health at the individual and community levels, providing health promotion, protection, disease prevention, diagnosis, treatment, rehabilitation, and maintenance. Services are organized by teams and directed toward populations within specific territories^(15)^.

In 1998, the federal government established the payment base fund (PAB) for PHC, consisting of a fixed per capita amount based on the municipal population and a variable component tied to federal priorities. The variable component incentivized municipalities to adopt the FHS model. While PAB transfers helped expand FHS, municipalities continued to co-finance PHC services, provide infrastructure and supplies, and hire staff. In 2019, the Prevent Brazil policy replaced the PAB, and with this new act, the per capita payment per municipality resident changed. Instead, payments are now based on people’s registrations with FHS teams, adjusted for socioeconomic vulnerability, and performance-based incentives for teams have been introduced^(16)^.

Family health teams in Brazil typically include one general physician, one registered nurse, four nursing technicians, and one community health worker (CHW), and they are intended to cover 4,000 residents^(14)^. Currently, approximately 70% of the Brazilian population is covered by FHS units^(8)^. Services offered to enrolled patients include consultations with physicians and nurses, minor acute care, health promotion, disease prevention, group education, vaccinations, and procedures such as medication administration, wound dressing, and blood sampling, among others. Close collaboration and frequent information exchange among FHS teams are needed. Nurses work autonomously and simultaneously collaborate with physicians, e.g., in cases where prescriptions are required^(15)^.

#### Nursing History

Formal nursing education in Brazil began in major cities with institutions such as the Alfredo Pinto School (1890) in Rio de Janeiro, the School of Nurses of Hospital Samaritano (1901) in São Paulo, and the Red Cross School (1916) in Rio de Janeiro^(17)^. In 1920, Brazil established the National Department of Public Health (DNSP) to manage and develop the health care system. Within this department, the first state nursing school, the Nursing School of the DNSP, was founded in 1922 and renamed the Anna Néry School in 1926^(18)^. The education of the first nurses in Brazil, led by American nurses, was based on an adaptation of Florence Nightingale’s model^(18)^.

In 1949, a federal law established regulations for nursing schools, requiring them to be affiliated with a university center or medical faculty, thereby elevating nursing education to the level of a university diploma. Additionally, 18-month programs for nursing auxiliaries were introduced under Law number 775/49 to address the shortage of skilled health care workers (Lei 775/49). By the end of the 1960s, thirty-two nursing programs were established at universities in Brazil. Furthermore, in 1968, a university reform was enacted, changing the structure of the programs, including those for nursing studies, and limiting the designation of higher education to universities^(19)^. Overall, the professionalization of nurses at the university level in Brazil began experimentally in the early 1930s, increased around the 1950s, and was officially established by the state in 1968.

The growing number of nurses led to the foundation of the Federal Council of Nursing (COFEN) and 27 Regional Nursing Councils, one for each state and Brasília (federal district), in 1973-a structure still in use today. Membership is mandatory for all nursing personnel. A significant milestone was the 1986 nursing professional practice law, which continues to regulate the profession. This law also defines advanced nursing tasks, including prescription rights for nurses in public health programs and routine care, if they are approved by a health institution^(20)^.

#### Nursing Education

In 1962, nurses gained academic recognition, at which point the title of nurse was equal with other higher education degrees^(21)^. Universities established nursing faculties to meet service demands, including training for management roles. The 1968 University Reform (Lei nº 5.540/1968) standardized nursing education with a common core and three specialties: obstetric nursing, public health nursing, and medical-surgical nursing^(21)^. Additionally, bachelor’s programs began offering the option to qualify as a licensed nurse.

These educational reforms opened pathways for nurses to pursue postgraduate studies, including both lato sensu (specializations and residency) and stricto sensu (master’s and doctoral) programs. Lato sensu specializations cover diverse nursing fields and require at least 360 study hours^(22)^. Additionally, specialized residency programs were introduced in 2005 and were offered as either uniprofessional or multiprofessional programs, with a total of 5,760 hours-80% dedicated to practical training and 20% to theoretical coursework^(23)^.

Stricto sensu education encompasses master’s and doctoral programs regulated by Opinion number 7/2017, with two types of master’s programs: academic, which prepares students for research, and professional, focused on applying advanced knowledge to real-world challenges^(24)^. In 2024, a residency program linked to a master’s course was approved by CAPES^(25)^.

The National Curricular Guidelines (NCGs) for higher education, approved in 2001, replaced the concept of a minimum curriculum in nursing. However, the curriculum remains fragmented, with a disconnect between general and specialized training, which runs counter to the principle of comprehensive learning that should ideally guide health education in Brazil^(26)^. A bachelor’s degree in nursing requires a minimum of 4,000 hours over at least five years, with faculty members determining the distribution of hours, content, and practical learning settings. Currently, nursing represents 57% of all health care personnel and are widely employed in PHC and hospitals across both the public and private sector^(8)^.

#### Advanced Practice Nursing

In the Brazilian context, APN has not yet been widely implemented. However, a notable event marked the beginning of APN discussions: a meeting in November 2015 at the Pan American Health Organization (PAHO) headquarters in Washington DC, USA, attended by representatives of the Brazilian Association of Nursing and COFEN, among other nursing representatives. Key objectives included re-evaluating the terminology for these nursing roles, considering the integration of APN into Brazil’s SUS, and examining the contributions of nursing to PHC within broader public policies^(27)^.

In response to the 2015 meeting, COFEN and its regional councils launched an APN committee in 2016 to explore and advance APN implementation in Brazil. This committee has contributed significantly to APN development through initiatives such as regulatory updates, concept clarification, educational policy proposals, and the funding of APN research^(28)^.

In 2016, Brazilian journals began publishing research on APN conducted by Brazilian nurses. Initial publications included studies on nurse practitioners in the English National Health Service, describing APN development in that context^(29)^. Subsequent articles suggested strategies for APN implementation in Brazil, emphasizing investment in professional education, national strategies for continuing education, expansion of evidence-based practices, and revisions to legislation supporting expanded practice^(30)^.

Building on recent advancements, instruments for evaluating nursing work and identifying APN competencies have been translated into Portuguese in validation studies^(31,32)^. These studies indicate that PHC nurses currently perform both clinical and nonclinical competencies, albeit partially. By 2024, the CAPES database listed 25 theses and dissertations on APN, representing advances on research about the theme.

In alignment with these developments, the Ministry of Health, in 2023, established a workgroup with various nursing organizations to further discuss APN, focusing on specialized training (residency programs), the regulation of professional practice, labor relations, and the standardization of APN titles and concepts ^(33)^. In this process, APN implementation in Brazil is recognized as a social process that should incorporate SUS principles, highlighting SUS users’ participation in the development and introduction of this new practice.

### The German Case

#### Characteristics of Primary Health Care

In Germany, PHC is delivered within a social health insurance system and is predominantly provided by GPs working in single or group practices. Since GPs are not considered gatekeepers of the system, patients are free to choose their GP. Nursing and therapeutic services (e.g., physiotherapy) need to be prescribed by GPs or other physicians. Patients can directly access a variety of services, e.g., specialist physicians, psychotherapy, and dentistry, which are covered by health insurance funds^(3)^.

Opportunities for multiprofessional, team-based PHC, e.g., psychotherapists, nurses, physiotherapists and occupational therapists, remain limited^(34)^. However, research has underscored the need to improve the skill mix and coordination of services to strengthen PHC, particularly in rural areas facing GP shortages and an increasing number of older patients with chronic illness; furthermore, the use of interprofessional care approaches is advantageous^(3,35)^.

At the intersection of PHC and long-term care, nurses already play a significant role in PHC provision. In this context, nursing teams provide treatment care, basic personal care, household assistance, and palliative care. However, prevention and health promotion remain underrepresented compared with rehabilitation and curative services^(3)^.

New nursing roles in PHC beyond classical home care services have been promoted, in which nurses assume, for example, patient education and counseling; preventive consultations with patients; and clinical tasks such as blood pressure control, case management and social support^(36)^. Nonetheless, challenges include overcoming traditional professional hierarchies and advancing the academic training of nurses and other health professionals^(35)^. Ongoing initiatives, including political initiatives, aim to expand nurses’ roles. Early studies have shown that expanding nurses’ roles in PHC in Germany can improve health care provision^(37)^.

#### Nursing History

Nursing in Germany has its origins in Christian altruism traditions, which Hilde Steppe^(38)^ describes as “textbook care for [the] gender specific division of labor”. This foundation continues to influence nurses’ autonomy in Germany. A significant milestone was the development of wartime nursing care during World War I (1914-1918). The first Nursing Act, passed in 1938, established federal regulations for nursing training, supporting the recruitment of future nurses^(39)^. During World War II, the high demand for nursing care led to the deployment of auxiliary nurses, who had received only minimal training^(40)^.

The 1957 revision of the 1938 Nursing Act introduced pediatric nursing as a second nursing profession alongside general nursing and established the first guidelines for auxiliary nurses^(40,41)^. To date, auxiliary nurses remain trained health care professionals who provide basic health care under nurse supervision. Additionally, in the 1960s, “gerontological nursing” was introduced as a third nursing profession^(42)^. Federal regulation of gerontological nursing education was first established in 2003 with the Gerontological Nursing Care Act.

In 2004, the Federal Ministry of Health (BMG) introduced a new Nursing Act that adopted a health-oriented approach for general and pediatric nursing. The act emphasized that nursing care should focus on “the recovery, improvement, maintenance, and promotion of physical and mental health” (§ 3 Abs. 1)^(43)^. The aim of this act was to strengthen nursing as an independent profession rather than a subordinate role to physicians. In the most recent educational reform, the Nursing Professions Act in 2020, the three separate nursing professions-general, pediatric and gerontological-were unified into a single profession, now titled “nursing specialist”^(44)^.

Nurses’ interests are represented by various stakeholders, such as the German Nurses Association and the German Nursing Council, although these organizations hold limited political influence. While initial steps have been taken, progress toward implementing nursing boards at the state and federal levels in Germany remains limited^(45)^.

#### Nursing Education

Historically, German nursing education has been predominantly a vocational pathway-school-based, physician-supervised, and state-regulated^(46)^. Today, nursing education in Germany follows a dual structure: the dominant route remains vocational education and training, but academic programs are becoming increasingly important. The most common path remains the state-regulated three-year vocational program, which was most recently reformed under the Nursing Professions Act (in force since 2020). This law introduced generalist nursing education, replacing previously separate training tracks for general, pediatric, and geriatric nursing with a unified curriculum that allows graduates to work across all areas of care. Since the 1990s, efforts to academize nursing alongside vocational training have increased. Initially, nursing pedagogy study programs were developed to train teachers in occupational nursing schools. By the 2000s, academic programs in nursing education and in nursing management expanded significantly at universities of applied sciences^(47)^. After the Bologna Accords, these programs were restructured into bachelor’s and master’s degrees in the late 2000s.

Since the 2010s, more academic programs have been introduced to offer primary qualifying bachelor’s degrees in nursing, enabling graduates to practice as nurses^(48)^. However, at the postgraduate level, most master’s programs continue to focus on education or management rather than on advanced clinical practice^(49)^. Despite ongoing efforts to promote nursing academization, only approximately 1 % of new entrants to the profession choose a primary qualifying bachelor’s program, and only 2.3 % pursue any type of nursing-related academic degree^(50)^.

#### Advanced Practice Nursing

In Germany, APN development is in an early and still evolving phase of institutionalization and can be described as having two partly overlapping tracks: broader reforms to expand nursing competencies in general and APN initiatives that are more specific. On the APN-specific track, since the late 2000s, a few postgraduate programs have been established to prepare nurses for advanced practice roles, yet graduates are still rare in clinical settings^(51)^. According to CHE Zeit University Ranking, only a few APN master’s programs exist in Germany (https://studiengaenge.zeit.de/studienangebote). However, they show significant heterogeneity, particularly regarding practice training hours and content^(3)^. The available evidence suggests that many APN graduates currently work in hospitals rather than in PHC^(49,52)^. Recent studies addressing APN in PHC in Germany indicate that APNs can help address service gaps, provide more continuous patient support, and relieve GPs by taking over routine tasks and offering collegial feedback (e.g. Büchler et al)^(53)^.

On the broader reform track, several legal initiatives have aimed to strengthen nursing in general-including efforts to advance the academization of the profession-rather than focusing solely on APN. Historically, nurses’ activities have largely depended on physicians’ prescriptions, reinforcing a hierarchical relationship between the professions. In 2008, the Nursing Further Development Act and subsequent regulations created a legal basis for enabling further qualified nurses to provide selected medical services within time-limited model projects-particularly in the areas of diabetes management, chronic wound care and dementia care (initially under § 63 Abs. 3b/3c SGB V and later reinforced by § 64d SGB V). However, implementation remained limited, and many projects were discontinued once funding ended. More recently, the Nursing Study Strengthening Act specifies competencies for the independent provision of extended clinical services in diabetes management, chronic wound care and dementia care, grounded in scientific methodology and evidence-based practice^(54)^. A more decisive shift is marked by the Act on Expanding Nursing Responsibilities and Reducing Bureaucracy in Nursing Care, which is currently under legislative development and is expected to be implemented in 2026; this act, for the first time, authorizes general nurses, as well as those with specialized advanced qualifications, to provide selected medical services autonomously within regular care pathways^(55)^.

In addition, an APN law has been announced, which has been under development for the past two years. The aim of the APN Act is to define a master’s-level APN qualification, protect the APN title and specify a scope of practice that enables APNs to independently provide selected medical services within regular care (BT-Drs. 20/14988). Notably, the initiatives mentioned above do not predominantly or explicitly address the implementation of APN roles in PHC-a gap already highlighted by the DBfK, which points to Community Health Nurses (CHN) which are qualified at masters level as performing not only expanded clinical care practices but a more holistic PHC approach in line with WHO recommendations^(54)^.

## DISCUSSION

In Brazil and Germany, the first steps have been taken to establish APN in PHC; however, these two countries have taken these steps in the context of different-sometimes very different-starting points in terms of nursing history, nursing education, nursing professionalization and the PHC model. Within this context, we reflect on how these differences may shape early APN pathways in PHC in both countries, pointing to initial lessons to be learned.

### Professional Identity and Early Academic Integration as Drivers of Advanced Practice Nursing in Primary Health Care

The ongoing development of APN is closely tied to the historical positioning and professional identity of nursing within each country. In Brazil, the early integration of nursing into the university system^(19,21)^ and its long-standing presence in public health have contributed to a more autonomous and academically grounded professional role. Nurses are highly visible in community-based PHC and frequently lead FHS teams^(15,56)^. This visibility provides fertile ground for advancing APN roles, even though formal implementation remains limited. Brazilian nurses already perform a wide range of clinical and coordinative tasks under institutional protocols-including prescribing in public health programs (Lei nº 7.498, 1986)^(20)^-which require competencies associated with advanced practice.

In contrast, the German nursing profession is still rooted in the tradition of subordination to medicine, with academic education introduced much later than in Brazil and in fragmented ways^(38,46)^. These historical legacies continue to shape perceptions of nursing and constrain the expansion of its role in PHC^(36,51)^. As comparative research shows, such historical trajectories create path dependencies: countries where nursing professionalized early-through academic integration and public health engagement-are better positioned to develop and embed advanced roles such as APN^(57,58)^.

### Education as a Foundation for Role Expansion

The organization of education is a crucial precondition for the sustainable implementation of APN, as the role typically requires a master’s degree as a formal qualification^(2)^. In Brazil, the well-established academic orientation of nursing education, with a strong emphasis on postgraduate training, including specializations, residencies, and professional master’s degrees^(22,23)^, represents a key enabling factor for APN development. Although formal legislation for APN roles has not been passed, this academic culture can provide a solid foundation for building advanced clinical expertise and role differentiation in nursing^(28)^.

On the other hand, the fragmentation of nursing undergraduate curricula in Brazil constitutes a considerable barrier to the development of advanced competencies^.^ APN education requires the integration of clinical knowledge, evidence-based decision-making, leadership, and care management-competencies that depend on coherent, longitudinal, and competency-based curricular structures^(59)^. Global frameworks emphasize that fragmented educational models, often organized into isolated disciplines with limited theory-practice integration, hinder the development of complex clinical reasoning and professional autonomy, both of which are essential for advanced practice^(2,60)^. In this context, curricular fragmentation restricts the articulation of knowledge and compromises nurses’ ability to provide comprehensive, autonomous, and high-quality care, thereby representing a key obstacle to the consolidation of APN within health systems.

In Germany, the delayed and fragmented academization of nursing education further poses a significant barrier to the development of APN. Although reforms such as the 2020 Nursing Professions Act have introduced generalist academic qualifications and laid the groundwork, relatively few nurses are currently pursuing academic degrees^(50)^. An additional obstacle lies in the current structure of most master’s programs, which predominantly focus on education or management rather than advanced clinical practice^(49)^. This, in turn, significantly limits the broader implementation of APN roles-particularly within the context of PHC.

### Primary Health Care Models as Structural Enablers or Barriers

The organizational model of PHC itself shapes the space for APN. Brazil’s multiprofessional PHC model actively involves nurses in population-based care, prevention, and health promotion-functions that naturally align with APN competencies. Germany’s PHC remains physician-centered, highly fragmented, and oriented toward curative care, with limited integration of nursing professionals outside home care settings. This reflects a broader, internationally observed pattern: the extent to which APNs can be implemented in PHC is strongly influenced by the structural and organizational features of the respective health system, particularly in the design of PHC, as, after all, PHC approaches are organized in diverse ways across countries^(61)^.

Beyond this structural foundation, the actual implementation of APN within PHC is also shaped by how well care models support interprofessional collaboration, role clarity, and shared clinical responsibility^(52)^. In Brazil, team-based care structures and defined catchment areas facilitate coordinated services and nurse autonomy within PHC teams. In Germany, the lack of multiprofessional care teams makes it challenging to develop integrated APN roles in PHC. International comparisons underscore that advanced roles can flourish only when PHC systems promote continuity of care, team-based decision-making, and mechanisms for clearly allocating responsibilities across professions^(1)^.

### Enabling Advanced Practice Nursing Implementation through Political Commitment

An additional consideration is the political will to support APN development. In Brazil, national actors such as the Ministry of Health and the COFEN, which also serves as an official representative body for nurses, have been mobilized to coordinate APN-related research and regulatory efforts, reflecting a growing degree of institutional alignment.

Germany’s recent legislative initiatives-such as the Nursing Competency Act-suggest political momentum but currently lack a coherent long-term strategy and a protected APN role definition. Notably, the limited political representation of the nursing profession-as exemplified by the absence or incomplete implementation of nursing boards-remains a concern. Stronger involvement of nurses is needed to meaningfully inform and co-shape APN development. In summary, in both countries, sustained political commitment can be seen as a key driver in establishing an enabling environment for APN integration, as emphasized by various studies (e.g., Torrens et al.)^(62)^.

### Brazil and Germany - Apples and Oranges? Why Contrasting Health Systems Offer Valuable Lessons for Advanced Practice Nursing Development

While Brazil and Germany differ substantially in terms of demographic, economic, and health system structures (see Square 1), which limits the direct comparability of APN implementation pathways, this contrast is analytically valuable. The diversity between the two contexts highlights how different institutional arrangements, professional traditions, and policies shape the development of APN in distinct ways. Instead of seeking symmetry, the comparison enables a broader understanding of the conditions under which APN roles emerge (or do not emerge) and the traditions that shape them. It allows for the identification of context-sensitive strategies and transferable lessons that may inspire innovation across diverse PHC systems.

### Limitations

Despite its informative character, this study also bears limitations. First, APN is still emerging in both countries; thus, the analysis relies on evolving rather than fully established role concepts. Second, language barriers also played a role, as some relevant documents-such as those on nursing history or national legislation-are partly available only in Portuguese or German. However, through the binational team exchange, this barrier could be effectively resolved. Finally, primary data capturing stakeholder perspectives were not included, which may have limited the depth of the analysis; however, related publications from the broader project address these perspectives.

### Contributions for nursing field

This comparative analysis of APN development in PHC in Brazil and Germany highlights that the likelihood of integrating APN into PHC is shaped by a complex interplay of professional, educational, institutional, and political conditions. Although steps toward expanding nursing roles in PHC have been taken in both countries, their trajectories reflect distinct starting points and systemic conditions. Brazil’s long-standing involvement of nurses in community-based PHC, combined with an already strong and growing academic infrastructure, provides a solid foundation for APN development, even as formal regulatory frameworks are still lacking. In Germany, recent legislative reforms and existing APN study programs signal a willingness to progress, yet structural challenges remain-particularly in education, academization and PHC organization.

This must be emphasized even more as evidence shows the relevance of APN for direct patient care, including improvement in care outcomes, particularly in the management of chronic conditions and access to health services. A systematic review^(61)^ shows that expanding nurses’ scope of practice is associated with reduced waiting times, greater problem-solving capacity in PHC, and clinical outcomes comparable to those achieved by physicians, along with high levels of patient satisfaction. APN effectively contributes to treatment adherence and continuity of care^(62)^. In this sense, organizational and educational differences across national contexts not only shape professional autonomy but also directly influence the quality, efficiency, and effectiveness of health systems.

## FINAL CONSIDERATIONS

APN implementation cannot be approached in isolation. Successfully implementing APN depends on the use of strategies that align educational reform, professional regulation, health system design, and political support. Rather than offering a one-size-fits-all model, this comparison underscores the value of context-sensitive approaches.

## Data Availability

Available research data is in the body of the article.
