# Supplementary material for: Advanced practice nursing role development in primary health care in Brazil and Germany: comparative perspective
Source: Rev Bras Enferm. 2026 Oct 16;79:e20250672. doi: 10.1590/0034-7167-2025-0672 (PMC13679170; doi:10.1590/0034-7167-2025-0672)
Supplement: Translation

# Translation: Desenvolvimento da Prática Avançada de Enfermagem na Atenção Primária no Brasil e Alemanha: perspectiva comparativa

**DOI:** 10.1590/0034-7167-2025-0672pt

**Authors:** Daria Andrea Bula, Kerstin Hämel, Elyne Montenegro Engstrom, Lígia Giovanella, Vanessa Cappellesso Horevicz Felix, Rosa Maria Rodrigues, Maria Lúcia Frizon Rizzotto, Marcus Heumann, Daniela Rosalba Luisi, Maike Zwergel, Beatriz Rosana Gonçalves de Oliveira Toso

**Affiliations:** I Bielefeld University. Bielefeld, North Westphalia, Germany; II University of Vienna. Vienna, Austria; III Fundação Oswaldo Cruz. Rio de Janeiro, Rio de Janeiro, Brazil; IV Universidade Estadual do Oeste do Paraná. Cascavel, Paraná, Brazil

**Keywords:** Prática Avançada de Enfermagem, Profissionais de Enfermagem, Atenção Primária à, Saúde, Papel do Profissional de Enfermagem, Enfermeira.

## Abstract

**Objetivo:**

Comparar o desenvolvimento da Prática Avançada de Enfermagem (PAE) na Atenção Primária à Saúde no Brasil e na Alemanha, focando em fatores institucionais, históricos e educacionais.

**Métodos:**

Estudo qualitativo comparativo baseado na análise de documentos de políticas, literatura e dados secundários sobre sistemas de saúde.

**Resultados:**

No Brasil, a formação acadêmica consolidada, as responsabilidades estabelecidas e os debates em curso favorecem o desenvolvimento da PAE, embora a ausência de regulamentação permaneça como barreira importante e persistente. Na Alemanha, o desenvolvimento reflete tradição técnico-profissional e recente academização, com poucos programas de mestrado em PAE. O modelo de atenção primária centrado no médico e limitações legais dificultam a implementação, apesar do crescente impulso político e institucional recente observado no país.

**Conclusões:**

O desenvolvimento da PAE depende do contexto, sem modelo único, devendo alinhar-se às estruturas institucionais, formação e papéis profissionais, exigindo estratégias adaptadas, sustentáveis e efetivas em diferentes níveis do sistema.

## INTRODUÇÃO

Diante do aumento da prevalência de doenças crônicas, do envelhecimento demográfico e da crescente pressão sobre a força de trabalho, muitos países intensificaram os esforços para fortalecer o papel dos enfermeiros na Atenção Primária à Saúde (APS). Evidências crescentes mostram que os enfermeiros contribuem significativamente para uma APS de alta qualidade, centrada no paciente e na comunidade, especialmente quando atuam em funções avançadas, como Enfermeiros de Prática Avançada (EPAs)^(1)^.

Os EPAs possuem tipicamente mestrado e combinam competências clínicas avançadas com habilidades de tomada de decisão autônoma e coordenação do cuidado^(2)^. Seu papel tem sido adotado em diversos países e é cada vez mais visto como uma estratégia-chave para enfrentar necessidades de saúde complexas e aumentar a capacidade da APS^(1)^. Além da prática clínica avançada, os EPAs também assumem responsabilidades em educação, liderança, pesquisa e desenvolvimento de serviços. No entanto, a integração dos enfermeiros na APS varia significativamente entre os sistemas de saúde, refletindo diferenças nos contextos institucionais, históricos e regulatórios^(2)^.

Por exemplo, no Brasil, os enfermeiros desempenham um papel fundamental na APS desde que a Estratégia Saúde da Família (ESF) estabeleceu um modelo multiprofissional de atenção primária. Em contraste, na Alemanha, a APS é predominantemente prestada por médicos generalistas (MGs) em consultórios privados, onde os enfermeiros não desempenham papel ou têm participação limitada^(3)^. Consequentemente, Brasil e Alemanha representam dois modelos fundamentalmente distintos de provisão de APS - um multiprofissional e inclusivo para a enfermagem, e outro liderado por médicos com participação restrita da enfermagem. No entanto, em ambos os países, estão em curso iniciativas com o objetivo de estabelecer o papel dos EPAs na APS.

Neste artigo, exploramos e comparamos “como o desenvolvimento da Prática Avançada de Enfermagem (PAE) na APS é moldado no Brasil e na Alemanha” e examinamos como fatores institucionais, históricos e educacionais influenciam as perspectivas de implementação da PAE. Para responder a essa questão de pesquisa, comparamos as histórias da enfermagem, as trajetórias educacionais e as iniciativas recentes para introduzir os papéis de EPAs na APS em ambos os países.

## OBJETIVO

Comparar o desenvolvimento da PAE na APS no Brasil e na Alemanha, focando em fatores institucionais, históricos e educacionais.

## MÉTODOS

### Aspectos éticos

A aprovação por comitê de ética não foi necessária para este tipo de estudo. Os pesquisadores seguiram o guia *Standards for Reporting Qualitative Research*, quando aplicável. Embora elementos do *COnsolidated criteria for REporting Qualitative research* (COREQ) tenham sido considerados para aumentar a clareza na descrição dos procedimentos qualitativos, o delineamento do estudo, baseado exclusivamente na análise documental, não se alinha totalmente aos domínios do COREQ, que são predominantemente voltados a pesquisas baseadas em entrevistas e grupos focais.

### Tipo de estudo e referencial teórico

O estudo foi realizado como parte do projeto de pesquisa “*Strengthening Advanced Practice Nursing and Collaboration in Primary Health Care* (APN-PHC)”. O objetivo do projeto foi identificar as forças, oportunidades e obstáculos da expansão dos papéis da enfermagem e avaliar como a PAE poderia contribuir para o avanço da APS no Brasil e na Alemanha. Foi adotado um delineamento qualitativo comparativo, adequado para examinar semelhanças e diferenças entre contextos nacionais distintos e para gerar interpretações aprofundadas e sensíveis ao contexto de fenômenos sociais e profissionais complexos. Essa abordagem é particularmente adequada para compreender como fatores estruturais, históricos e organizacionais moldam as práticas profissionais em diferentes contextos, permitindo análises intra e entre casos^(4)^.

Realizamos uma análise comparativa combinando busca documental e dados de fontes estatísticas (inter)nacionais (por exemplo, Organização para a Cooperação e Desenvolvimento Econômico, Organização Mundial da Saúde (OMS) e estatísticas nacionais). A literatura e os documentos incluíram políticas nacionais, marcos legais, publicações acadêmicas e relatórios de entidades profissionais no Brasil e na Alemanha. O desenvolvimento de estudos comparativos no campo da análise de políticas públicas pode ser considerado recente, remontando às décadas de 1960 e 1970, embora seja uma prática comum na ciência política desde seus primórdios^(4)^.

O Quadro 1 apresenta uma visão geral dos dados demográficos, indicadores de saúde e população, e da força de trabalho em saúde de ambos os países. A análise desses dados sob uma perspectiva comparativa permite posicionar diferentes condições contextuais lado a lado e aprender com as experiências de cada sistema de saúde. Ao fazê-lo, utilizamos uma abordagem comparativa amplamente empregada em pesquisas de políticas públicas e sistemas de saúde. Conforme destacado no *Bulletin of the WHO*, comparações internacionais das características dos sistemas de saúde podem fornecer evidências valiosas e subsidiar políticas de saúde^(5)^.

**Quadro 1:** Indicadores demográficos, econômicos e de saúde no Brasil e na Alemanha, 2025

Indicadores	Brasil	Alemanha
População	211.998.573 milhões (2024)	83.510.950 milhões (2024)
Taxa de crescimento populacional	0,4% (2024)	-0,5% (2024)
Área territorial	8.509.379,576 km^2^ (2024)	357.596 km^2^ (2022)
Densidade demográfica	25 habitantes/km^2^ (2023)	240 habitantes/km^2^ (2023)
Produto Interno Bruto	10.280,03 dólares americanos (2024)	55.800,02 dólares americanos (2024)
Expectativa de vida	76 (2023)	81 (2023)
Taxa de fertilidade	1,62 (2023)	1,39 (2023)
Taxa de mortalidade infantil por 1.000 nascidos vivos	12,5 (2023)	3,2 (2023)
Enfermeiros/1.000 habitantes	5,3 (2023)	12,25 (2023)
Salário de enfermagem	4.750 (bruto) reais/mês^*^	4,329 (bruto) euros/mês

Fonte:^(7-12)^

* Aprovada pela Lei 14.434/22^(13)^.

### Síntese das principais características dos países

Foram identificadas diferenças nos indicadores demográficos, econômicos e relacionados à saúde entre Brasil e Alemanha. A população do Brasil é significativamente maior (211.998.573 milhões vs. 83.510.950 milhões) e mais dispersa territorialmente, resultando em uma densidade populacional muito menor em comparação com a Alemanha (Quadro 1). Muitas regiões brasileiras, especialmente na Amazônia e em áreas rurais, são de difícil acesso, o que dificulta ainda mais a infraestrutura e a prestação de serviços de saúde^(6)^. Em contraste, a Alemanha apresenta níveis substancialmente mais altos de desenvolvimento econômico, refletidos no Produto Interno Bruto *per capita* (55.800,2 USD vs. 10.280,3 USD), maior expectativa de vida (81 vs. 76 anos) e menor mortalidade infantil (3,2 vs. 12,5 por 1.000 nascidos vivos). Além disso, a Alemanha possui maior densidade de profissionais de enfermagem (12,25 vs. 5,3 enfermeiros por 1.000 habitantes).

### Procedimentos metodológicos e análise de dados

Para facilitar as comparações entre Brasil e Alemanha, para a coleta de dados, desenvolvemos uma matriz (Quadro 2) que descreve dimensões-chave da APS e da enfermagem, abrangendo concepção, organização e serviços da APS, bem como história, educação e desenvolvimento da PAE. Esses foram os mesmos temas utilizados nas análises indutivas, de acordo com Meny e Thoenig^(4)^.

**Quadro 2:** Matriz de análise comparativa para sistemas de saúde, Atenção Primária à Saúde e Prática Avançada de Enfermagem, 2025

Características da Atenção Primária à Saúde		
**Concepção da Atenção Primária à Saúde** Qual é o conceito? Qual é o papel da Atenção Primária à Saúde no sistema de saúde?	**Modelo de atenção** Qual é a organização predominante da Atenção Primária à Saúde? Que tipos de serviços estão disponíveis na Atenção Primária à Saúde? Quais profissionais atuam na Atenção Primária à Saúde?	**Integração em rede** Qual é o papel da Atenção Primária à Saúde na rede de serviços de saúde?
**Características da enfermagem**		
**História da enfermagem** Quais são os marcos na história da enfermagem?	**Educação em enfermagem** Como se desenvolveu o ensino de enfermagem e qual é o estado atual desse ensino?	**Prática Avançada de Enfermagem** Em que medida a Prática Avançada de Enfermagem está estabelecida? Quais são os principais desenvolvimentos, incluindo os mais recentes?

### Cenário do estudo e casos

O estudo concentrou-se em dois contextos nacionais com sistemas de saúde e modelos de educação em enfermagem distintos, selecionados com base em sua relevância para o objetivo da pesquisa e em suas abordagens contrastantes em relação à PAE. Esse delineamento comparativo permite explorar como trajetórias históricas, arranjos institucionais e marcos de políticas influenciam a autonomia profissional e o escopo de prática.

### Fontes de dados e seleção de documentos

Os dados consistiram exclusivamente em fontes documentais, incluindo políticas nacionais de saúde, marcos regulatórios, diretrizes curriculares, padrões profissionais e relatórios institucionais relacionados à educação e à prática em enfermagem. Os documentos foram selecionados por meio de estratégias intencionais e baseadas em critérios, priorizando relevância, credibilidade e atualidade. Incluíram-se publicações oficiais, artigos revisados por pares e documentos emitidos por organizações nacionais e internacionais reconhecidas. O processo de seleção buscou garantir profundidade e diversidade suficientes de fontes para sustentar uma análise comparativa robusta.

### Análise de dados

A análise de dados seguiu uma abordagem estruturada, orientada por conceitos. Um referencial analítico foi desenvolvido, *a priori*, para assegurar um exame abrangente e sensível ao contexto da questão de pesquisa. Domínios centrais, como características da APS, história da enfermagem e educação em enfermagem, orientaram a organização dos dados. A literatura e os documentos relevantes foram alocados iterativamente nesses domínios e, quando necessário, discutidos pela equipe binacional para superar barreiras linguísticas e garantir interpretação precisa. Essa abordagem possibilitou comparações intra e entre casos, bem como a identificação de convergências, divergências e padrões explicativos entre os contextos^(4,5)^.

## RESULTADOS

### O caso brasileiro

#### Características da Atenção Primária à Saúde

No Brasil, a APS está inserida no Sistema Único de Saúde (SUS), um sistema universal financiado por impostos, estabelecido pela Constituição Federal de 1988. O SUS garante acesso à atenção integral e está estruturado com base nos princípios de descentralização, regionalização e participação social, sendo os municípios responsáveis pela organização e prestação dos serviços de APS^(14)^. Além da APS, o SUS também abrange atenção especializada e hospitalar, serviços de urgência e procedimentos de alto custo. No âmbito do SUS, os municípios supervisionam os serviços de APS prestados em unidades públicas. Desde 1994, um modelo de APS orientado à comunidade, a ESF, passou a complementar as unidades básicas tradicionais. A ESF aborda a saúde nos níveis individual e comunitário, oferecendo promoção da saúde, proteção, prevenção de doenças, diagnóstico, tratamento, reabilitação e manutenção. Os serviços são organizados por equipes e direcionados a populações em territórios específicos^(15)^.

Em 1998, o governo federal estabeleceu o Piso de Atenção Básica (PAB) para a APS, composto por um valor fixo *per capita* baseado na população municipal e por um componente variável vinculado às prioridades federais. O componente variável incentivou os municípios a adotarem o modelo da ESF. Embora as transferências do PAB tenham contribuído para a expansão da ESF, os municípios continuaram a cofinanciar os serviços de APS, fornecer infraestrutura e insumos e contratar profissionais. Em 2019, a política Previne Brasil substituiu o PAB e, com essa nova medida, a forma de pagamento *per capita* por habitante foi alterada. Em vez disso, os repasses passaram a se basear no número de pessoas cadastradas nas equipes da ESF, ajustados pela vulnerabilidade socioeconômica, além da introdução de incentivos baseados no desempenho das equipes^(16)^.

As equipes da ESF no Brasil geralmente incluem um médico generalista, um enfermeiro, quatro técnicos de enfermagem e um agente comunitário de saúde, sendo projetadas para cobrir 4.000 habitantes^(14)^. Atualmente, aproximadamente 70% da população brasileira é coberta por unidades da ESF^(8)^. Os serviços oferecidos aos usuários cadastrados incluem consultas com médicos e enfermeiros, atendimento de casos agudos leves, promoção da saúde, prevenção de doenças, educação em grupo, vacinação e procedimentos como administração de medicamentos, curativos e coleta de sangue, entre outros. É necessário que haja uma colaboração estreita e troca frequente de informações entre as equipes da ESF. Os enfermeiros atuam de forma autônoma e, ao mesmo tempo, colaboram com médicos, por exemplo, em casos em que são necessárias prescrições^(15)^.

#### História da enfermagem

A educação formal em enfermagem no Brasil teve início em grandes cidades com instituições como a Escola Alfredo Pinto (1890), no Rio de Janeiro, a Escola de Enfermeiros do Hospital Samaritano (1901), em São Paulo, e a Escola da Cruz Vermelha (1916), no Rio de Janeiro^(17)^. Em 1920, o Brasil criou o Departamento Nacional de Saúde Pública (DNSP) para organizar e desenvolver o sistema de saúde. Nesse contexto, foi fundada, em 1922, a primeira escola estatal de enfermagem, a Escola de Enfermagem do DNSP, que foi renomeada como Escola Anna Nery em 1926^(18)^. A formação das primeiras enfermeiras no Brasil, conduzida por enfermeiras americanas, baseou-se em uma adaptação do modelo de Florence Nightingale^(18)^.

Em 1949, uma lei federal estabeleceu regulamentações para as escolas de enfermagem, exigindo sua vinculação a um centro universitário ou faculdade de medicina, elevando a formação em enfermagem ao nível de diploma universitário. Além disso, programas de 18 meses para auxiliares de enfermagem foram introduzidos pela Lei nº 775/49 para suprir a escassez de trabalhadores qualificados na saúde. Ao final da década de 1960, 32 cursos de enfermagem estavam estabelecidos em universidades brasileiras. Ademais, em 1968, foi implementada uma reforma universitária que modificou a estrutura dos cursos, incluindo os de enfermagem, e restringiu a denominação de ensino superior às universidades^(19)^. De modo geral, a profissionalização da enfermagem em nível universitário no Brasil começou de forma experimental no início da década de 1930, expandiu-se na década de 1950 e foi oficialmente consolidada pelo Estado em 1968.

O aumento do número de enfermeiros levou à criação do Conselho Federal de Enfermagem (COFEN) e de 27 Conselhos Regionais de Enfermagem, um para cada estado e para o Distrito Federal, em 1973 - estrutura que permanece até hoje. A filiação é obrigatória para todos os profissionais de enfermagem. Um marco importante foi a Lei do Exercício Profissional de 1986, que continua regulamentando a profissão. Essa lei também define atividades avançadas de enfermagem, incluindo a prescrição por enfermeiros em programas de saúde pública e em rotinas assistenciais, desde que aprovadas por instituição de saúde^(20)^.

#### Educação em enfermagem

Em 1962, os enfermeiros obtiveram reconhecimento acadêmico, passando o título de enfermeiro a ser equivalente aos demais diplomas de ensino superior^(21)^. As universidades criaram faculdades de enfermagem para atender às demandas dos serviços, incluindo a formação para funções gerenciais. A Reforma Universitária de 1968 (Lei nº 5.540/1968) padronizou a educação em enfermagem com um núcleo comum e três especialidades: enfermagem obstétrica, enfermagem em saúde pública e enfermagem médico-cirúrgica^(21)^. Além disso, os cursos de bacharelado passaram a oferecer a possibilidade de qualificação como enfermeiro licenciado.

Essas reformas educacionais abriram caminhos para que os enfermeiros pudessem seguir estudos de pós-graduação, incluindo programas *lato sensu* (especializações e residências) e *stricto sensu* (mestrado e doutorado). As especializações *lato sensu* abrangem diversas áreas da enfermagem e exigem no mínimo 360 horas de estudo^(22)^. Além disso, programas de residência especializada foram introduzidos em 2005, podendo ser uniprofissionais ou multiprofissionais, com carga horária total de 5.760 horas, sendo 80% destinadas à prática e 20% ao conteúdo teórico^(23)^.

A formação *stricto sensu* compreende programas de mestrado e doutorado regulamentados pelo Parecer nº 7/2017, com dois tipos de mestrado: acadêmico, voltado à formação para pesquisa, e profissional, focado na aplicação de conhecimentos avançados a problemas do mundo real^(24)^. Em 2024, foi aprovado um programa de residência vinculado ao mestrado pela Coordenação de Aperfeiçoamento de Pessoal de Nível Superior (CAPES)^(25)^.

As Diretrizes Curriculares Nacionais para o ensino superior, aprovadas em 2001, substituíram o conceito de currículo mínimo na enfermagem. No entanto, o currículo ainda permanece fragmentado, com uma desconexão entre a formação geral e a especializada, o que vai contra o princípio da formação integral que deveria orientar a educação em saúde no Brasil^(26)^. O curso de graduação em enfermagem exige carga mínima de 4.000 horas ao longo de pelo menos cinco anos, cabendo às instituições definir a distribuição da carga horária, os conteúdos e os cenários de prática. Atualmente, a enfermagem representa 57% de todos os profissionais de saúde e está amplamente inserida tanto na APS quanto nos hospitais, nos setores público e privado^(8)^.

#### Prática Avançada de Enfermagem

No contexto brasileiro, a PAE ainda não foi amplamente implementada. No entanto, um evento importante marcou o início das discussões sobre o tema: uma reunião realizada em novembro de 2015 na sede da Organização Pan-Americana da Saúde, em Washington DC, EUA, com a participação de representantes da Associação Brasileira de Enfermagem e do COFEN, entre outros representantes da enfermagem. Entre os principais objetivos estavam a reavaliação da terminologia desses papéis, a consideração da integração da PAE ao SUS e a análise das contribuições da enfermagem para a APS no âmbito das políticas públicas^(27)^.

Em resposta à reunião de 2015, o COFEN e seus conselhos regionais criaram, em 2016, uma comissão de PAE para explorar e promover sua implementação no Brasil. Essa comissão tem contribuído significativamente para o desenvolvimento da PAE por meio de iniciativas como atualização regulatória, esclarecimento conceitual, propostas de políticas educacionais e financiamento de pesquisas sobre PAE^(28)^.

Em 2016, periódicos brasileiros passaram a publicar pesquisas sobre PAE conduzidas por enfermeiros brasileiros. As publicações iniciais incluíram estudos sobre *nurse practitioners* (enfermeiros de prática avançada) no *National Health Service*, sistema de saúde inglês, descrevendo o desenvolvimento da PAE naquele contexto^(29)^. Estudos posteriores sugeriram estratégias para implementação da PAE no Brasil, enfatizando investimentos na formação profissional, estratégias nacionais de educação permanente, ampliação de práticas baseadas em evidências e revisão da legislação que sustenta a ampliação do escopo de prática^(30)^.

Com base em avanços recentes, instrumentos para avaliação do trabalho em enfermagem e identificação de competências em PAE foram traduzidos para o português em estudos de validação^(31,32)^. Esses estudos indicam que enfermeiros da APS já desempenham competências clínicas e não clínicas, ainda que de forma parcial. Em 2024, a base de dados da CAPES registrava 25 teses e dissertações sobre PAE, indicando avanços na produção científica sobre o tema.

Em consonância com esses avanços, o Ministério da Saúde, em 2023, instituiu um grupo de trabalho com diversas organizações de enfermagem para aprofundar as discussões sobre PAE, com foco na formação especializada (programas de residência), na regulamentação do exercício profissional, nas relações de trabalho e na padronização de títulos e conceitos relacionados à PAE^(33)^. Nesse processo, a implementação da PAE no Brasil é reconhecida como um processo social que deve incorporar os princípios do SUS, destacando a participação dos usuários no desenvolvimento e na introdução dessa nova prática.

### O caso alemão

#### Características da Atenção Primária à Saúde

Na Alemanha, a APS é prestada dentro de um sistema de seguro de saúde social e é predominantemente oferecida por MGs que atuam em consultórios individuais ou em grupo. Como os MGs não são considerados porta de entrada do sistema, os pacientes têm liberdade para escolher seu MG. Os serviços de enfermagem e terapêuticos (por exemplo, fisioterapia) precisam ser prescritos por MGs ou outros médicos. Os pacientes podem acessar diretamente diversos serviços, como especialistas, psicoterapia e odontologia, os quais são cobertos pelos fundos de seguro de saúde^(3)^.

As oportunidades para uma APS multiprofissional e baseada em equipes - por exemplo, envolvendo psicoterapeutas, enfermeiros, fisioterapeutas e terapeutas ocupacionais - ainda são limitadas^(34)^. No entanto, pesquisas têm destacado a necessidade de melhorar a composição de competências e a coordenação dos serviços para fortalecer a APS, especialmente em áreas rurais que enfrentam escassez de MG e um número crescente de pacientes idosos com doenças crônicas. Além disso, o uso de abordagens de cuidado interprofissional mostra-se vantajoso^(3,35)^.

Na interface entre APS e cuidados de longa duração, os enfermeiros já desempenham um papel significativo na prestação de cuidados. Nesse contexto, equipes de enfermagem oferecem cuidados terapêuticos, cuidados pessoais básicos, assistência domiciliar e cuidados paliativos. No entanto, ações de prevenção e promoção da saúde permanecem sub-representadas em comparação com serviços de reabilitação e curativos^(3)^.

Novos papéis da enfermagem na APS, além dos serviços tradicionais de cuidado domiciliar, vêm sendo promovidos, nos quais os enfermeiros assumem, por exemplo, educação e aconselhamento aos pacientes, consultas preventivas e tarefas clínicas, como controle da pressão arterial, gerenciamento de casos e suporte social^(36)^. Ainda assim, persistem desafios, incluindo a superação de hierarquias profissionais tradicionais e o avanço da formação acadêmica dos enfermeiros e de outros profissionais de saúde^(35)^. Iniciativas em andamento, incluindo políticas públicas, buscam expandir o papel dos enfermeiros. Estudos iniciais demonstram que essa expansão na APS alemã pode melhorar a prestação de cuidados de saúde^(37)^.

#### História da enfermagem

A enfermagem na Alemanha tem suas origens em tradições de altruísmo cristão, que Hilde Steppe^(38)^ descreve como “um exemplo clássico da divisão sexual do trabalho”. Essa base continua a influenciar a autonomia dos enfermeiros no país. Um marco importante foi o desenvolvimento do cuidado de enfermagem em contextos de guerra durante a Primeira Guerra Mundial (1914-1918). A primeira lei de enfermagem, promulgada em 1938, estabeleceu regulamentações federais para a formação em enfermagem, apoiando o recrutamento de novos profissionais^(39)^. Durante a Segunda Guerra Mundial, a alta demanda por cuidados levou à utilização de auxiliares de enfermagem com treinamento mínimo^(40)^.

A revisão de 1957 da Lei de Enfermagem de 1938 introduziu a enfermagem pediátrica como uma segunda profissão de enfermagem, ao lado da enfermagem geral, e estabeleceu as primeiras diretrizes para auxiliares de enfermagem^(40,41)^. Até hoje, os auxiliares permanecem como profissionais de saúde treinados que prestam cuidados básicos sob supervisão de enfermeiros. Além disso, na década de 1960, foi introduzida a “enfermagem gerontológica” como uma terceira profissão^(42)^. A regulamentação federal da formação em enfermagem gerontológica foi estabelecida pela primeira vez em 2003 com a Lei de Cuidados de Enfermagem Gerontológica (*Gerontological Nursing Care Act*).

Em 2004, o *Bundesministerium für Gesundheit* (Ministério Federal da Saúde) introduziu uma nova lei de enfermagem que adotou uma abordagem orientada à saúde para a enfermagem geral e pediátrica. A lei enfatizou que o cuidado de enfermagem deve se concentrar na “recuperação, melhoria, manutenção e promoção da saúde física e mental” (§ 3 *Absatz* 1)^(43)^. O objetivo dessa legislação foi fortalecer a enfermagem como profissão independente, em vez de subordinada à medicina. Na reforma educacional mais recente, a Lei das Profissões de Enfermagem (*Nursing Professions Act*) de 2020 unificou as três profissões - geral, pediátrica e gerontológica - em uma única, denominada “enfermeiro especialista”^(44)^.

Os interesses dos enfermeiros são representados por diferentes entidades, como a Associação Alemã de Enfermeiros (*German Nurses Association*) e o Conselho Alemão de Enfermagem (*German Nursing Council*), contudo essas organizações têm influência política limitada. Embora tenham sido dados passos iniciais, o progresso na implementação de conselhos de enfermagem em nível estadual e federal na Alemanha ainda é restrito^(45)^.

#### Educação em enfermagem

Historicamente, a educação em enfermagem na Alemanha tem sido predominantemente baseada em formação profissional técnica, realizada em escolas, supervisionada por médicos e regulamentada pelo Estado^(46)^. Atualmente, a formação segue uma estrutura dual: a via predominante ainda é a formação profissional técnica, mas os programas acadêmicos vêm ganhando importância. O caminho mais comum continua sendo o programa técnico de três anos regulamentado pelo Estado, recentemente reformulado pela Lei das Profissões de Enfermagem (em vigor desde 2020). Essa lei introduziu a formação generalista, substituindo os antigos percursos separados (geral, pediátrico e geriátrico) por um currículo unificado que permite atuação em todas as áreas do cuidado. Desde a década de 1990, os esforços para academização da enfermagem, paralelamente à formação técnica, têm aumentado. Inicialmente, foram desenvolvidos cursos de pedagogia em enfermagem para formar docentes das escolas técnicas. Na década de 2000, programas acadêmicos em educação e gestão em enfermagem se expandiram significativamente nas universidades de ciências aplicadas^(47)^. Após os acordos de Bolonha, esses programas foram reestruturados em cursos de bacharelado e mestrado no final dos anos 2000.

Desde a década de 2010, mais programas acadêmicos passaram a oferecer graduação em enfermagem com formação inicial, permitindo que os graduados atuem como enfermeiros^(48)^. No entanto, em nível de pós-graduação, a maioria dos programas de mestrado ainda se concentra em educação ou gestão, em vez de prática clínica avançada^(49)^. Apesar dos esforços contínuos para promover a academização, apenas aproximadamente 1% dos ingressantes optam por um bacharelado como formação inicial, e apenas 2,3% seguem qualquer tipo de formação acadêmica em enfermagem^(50)^.

#### Prática Avançada de Enfermagem

Na Alemanha, o desenvolvimento da PAE encontra-se em uma fase inicial e ainda em evolução de institucionalização, podendo ser descrito como composto por duas trajetórias parcialmente sobrepostas: reformas mais amplas para expansão das competências da enfermagem e iniciativas específicas da PAE. Na trajetória específica da PAE, desde o final dos anos 2000, alguns programas de pós-graduação foram criados para preparar enfermeiros para funções avançadas, embora os egressos ainda sejam pouco presentes nos contextos clínicos^(51)^. De acordo com o *CHE University Ranking*, existem poucos programas de mestrado em PAE na Alemanha (https://studiengaenge.zeit.de/studienangebote). No entanto, apresentam uma heterogeneidade significativa, particularmente no que diz respeito às horas de treino prático e ao conteúdo^(3)^. As evidências disponíveis sugerem que muitos desses profissionais atuam atualmente em hospitais, e não na APS^(49,52)^. Estudos recentes sobre EPAs na APS na Alemanha indicam que esses profissionais podem contribuir para suprir lacunas assistenciais, oferecer acompanhamento mais contínuo aos pacientes e aliviar a carga dos MGs ao assumir tarefas rotineiras e fornecer suporte clínico (por exemplo, Büchler *et al*.)^(53)^.

Na trajetória mais ampla de reformas, diversas iniciativas legais têm buscado fortalecer a enfermagem como um todo - incluindo a promoção da academização - e não exclusivamente a PAE. Historicamente, as atividades dos enfermeiros têm dependido amplamente de prescrições médicas, reforçando uma relação hierárquica entre as profissões. Em 2008, a Lei de Desenvolvimento da Enfermagem e regulamentações subsequentes criaram uma base legal para que enfermeiros com formação adicional pudessem prestar determinados serviços médicos em projetos-piloto com duração limitada, especialmente nas áreas de diabetes, cuidados com feridas crônicas e demência (inicialmente sob § 63 *Absatz* 3b/3c *Sozialgesetzbuch* (SGB) V e posteriormente reforçado pelo § 64d SGB V). Mais recentemente, a Lei de Fortalecimento dos Estudos em Enfermagem definiu competências para a prestação independente de serviços clínicos ampliados nas áreas de diabetes, feridas crônicas e demência, com base em metodologia científica e prática baseada em evidências^(54)^. Uma mudança mais decisiva é representada pela Lei de Expansão das Responsabilidades da Enfermagem e Redução da Burocracia no Cuidado, atualmente em desenvolvimento legislativo e com previsão de implementação em 2026. Essa lei autoriza, pela primeira vez, enfermeiros gerais e aqueles com qualificação avançada a prestarem determinados serviços médicos de forma autônoma dentro dos fluxos regulares de cuidado^(55)^.

Além disso, uma lei específica de PAE está em desenvolvimento há cerca de dois anos. Seu objetivo é definir uma qualificação em nível de mestrado, proteger o título de PAE e estabelecer um escopo de prática que permita EPAs prestar determinados serviços médicos de forma independente no cuidado regular. Notavelmente, as iniciativas mencionadas não abordam predominantemente a implementação da PAE na APS - uma lacuna já destacada pelo *Deutscher Berufsverband für Pflegeberufe*, que aponta que enfermeiros comunitários com mestrado desempenham não apenas práticas clínicas ampliadas, mas também uma abordagem mais holística da APS, alinhada às recomendações da OMS^(54)^.

## DISCUSSÃO

No Brasil e na Alemanha, os primeiros passos para a implementação da PAE na APS já foram dados; contudo, esses dois países deram esses passos partindo de pontos de partida diferentes - por vezes muito distintos - em termos de história da enfermagem, formação em enfermagem, profissionalização da enfermagem e modelo da APS. Nesse contexto, refletimos sobre como essas diferenças podem moldar os primeiros caminhos da PAE na APS em ambos os países, apontando lições iniciais a serem aprendidas.

### Identidade profissional e integração acadêmica precoce como impulsionadores da Prática Avançada de Enfermagem na Atenção Primária à Saúde

O desenvolvimento contínuo da PAE está intimamente relacionado ao posicionamento histórico e à identidade profissional da enfermagem em cada país. No Brasil, a integração precoce da enfermagem ao sistema universitário^(19,21)^ e sua presença consolidada na saúde pública contribuíram para um papel profissional mais autônomo e com base acadêmica sólida. Os enfermeiros são altamente visíveis na APS comunitária e frequentemente lideram equipes da ESF^(15,56)^. Essa visibilidade cria um ambiente favorável para o avanço da PAE, embora sua implementação formal ainda seja limitada. Os enfermeiros brasileiros já desempenham uma ampla gama de atividades clínicas e de coordenação sob protocolos institucionais, incluindo prescrição em programas de saúde pública (Lei nº 7.498, 1986)^(20)^, que exigem competências associadas à prática avançada.

Em contraste, a enfermagem alemã ainda está enraizada na tradição de subordinação à medicina, com a educação acadêmica introduzida muito mais tardiamente do que no Brasil e de forma fragmentada^(38,46)^. Esses legados históricos continuam a moldar as percepções sobre a profissão e limitam a expansão de seu papel na APS^(36,51)^. Como demonstram estudos comparativos, essas trajetórias históricas criam dependências de trajetória: países em que a enfermagem se profissionalizou precocemente - por meio da integração acadêmica e da atuação em saúde pública - estão mais bem posicionados para desenvolver e consolidar funções avançadas como a PAE^(57,58)^.

### Educação como base para a expansão de papéis

A organização da educação é uma condição fundamental para a implementação sustentável da PAE, uma vez que esse papel geralmente exige formação em nível de mestrado como qualificação formal^(2)^. No Brasil, a forte orientação acadêmica da educação em enfermagem, com ênfase na pós-graduação, incluindo especializações, residências e mestrados profissionais^(22,23)^, constitui um fator facilitador importante para o desenvolvimento da PAE. Embora ainda não exista legislação específica para a PAE, essa base acadêmica oferece condições sólidas para o desenvolvimento de *expertise* clínica avançada e diferenciação de papéis na enfermagem^(28)^.

Por outro lado, a fragmentação dos currículos de graduação em enfermagem no Brasil representa uma barreira relevante para o desenvolvimento de competências avançadas. A formação em PAE exige a integração de conhecimento clínico, tomada de decisão baseada em evidências, liderança e gestão do cuidado - competências que dependem de estruturas curriculares coerentes, longitudinais e baseadas em competências^(59)^. Modelos globais destacam que currículos fragmentados, frequentemente organizados em disciplinas isoladas com limitada integração teoria-prática, dificultam o desenvolvimento do raciocínio clínico complexo e da autonomia profissional, ambos essenciais para a prática avançada^(2,60)^. Nesse contexto, a fragmentação curricular limita a articulação do conhecimento e compromete a capacidade dos enfermeiros de oferecer cuidado abrangente, autônomo e de alta qualidade, constituindo um obstáculo importante à consolidação da PAE nos sistemas de saúde.

Na Alemanha, a academização tardia e fragmentada da enfermagem também representa uma barreira significativa ao desenvolvimento da PAE. Embora reformas como a Lei das Profissões de Enfermagem de 2020 tenham introduzido qualificações acadêmicas generalistas e estabelecido bases importantes, relativamente poucos enfermeiros seguem formação acadêmica^(50)^. Um obstáculo adicional reside na estrutura atual da maioria dos programas de mestrado, que se concentram predominantemente em educação ou gestão, em vez de prática clínica avançada^(49)^. Isso limita significativamente a implementação mais ampla da PAE, especialmente no contexto da APS.

### Modelos de Atenção Primária à Saúde como facilitadores ou barreiras estruturais

O próprio modelo organizacional da APS molda o espaço para a PAE. O modelo multiprofissional brasileiro envolve ativamente enfermeiros no cuidado populacional, na prevenção e na promoção da saúde - funções que se alinham naturalmente às competências da PAE. Já a APS alemã permanece centrada no médico, altamente fragmentada e orientada ao cuidado curativo, com limitada integração de profissionais de enfermagem fora do contexto de cuidados domiciliares. Esse cenário reflete um padrão observado internacionalmente: a possibilidade de implementação da PAE na APS é fortemente influenciada pelas características estruturais e organizacionais do sistema de saúde, especialmente pelo desenho da APS^(61)^.

Além dessa base estrutural, a implementação efetiva da PAE na APS também depende do grau em que os modelos de cuidado favorecem a colaboração interprofissional, a clareza de papéis e a responsabilidade clínica compartilhada^(52)^. No Brasil, estruturas de cuidado baseadas em equipes e territórios definidos facilitam a coordenação dos serviços e a autonomia dos enfermeiros dentro das equipes da APS. Na Alemanha, a ausência de equipes multiprofissionais consolidadas dificulta o desenvolvimento de papéis integrados de EPAs na APS. Comparações internacionais indicam que funções avançadas só se consolidam quando os sistemas de APS promovem continuidade do cuidado, tomada de decisão compartilhada e mecanismos claros de distribuição de responsabilidades entre profissões^(1)^.

### Viabilizando a implementação da Prática Avançada de Enfermagem por meio do compromisso político

Outro aspecto relevante é a vontade política de apoiar o desenvolvimento da PAE. No Brasil, atores nacionais como o Ministério da Saúde e o COFEN - que também atua como órgão representativo da enfermagem - têm se mobilizado para coordenar pesquisas e esforços regulatórios relacionados à PAE, refletindo um crescente alinhamento institucional.

Na Alemanha, iniciativas legislativas recentes, como leis voltadas à ampliação das competências da enfermagem, indicam um movimento político favorável, mas ainda carecem de uma estratégia coerente de longo prazo e de uma definição formal protegida para a PAE. Destaca-se também a limitada representação política da enfermagem, evidenciada pela ausência ou implementação incompleta de conselhos profissionais. É necessária uma maior participação dos enfermeiros para contribuir efetivamente na formulação e condução do desenvolvimento da PAE. Em síntese, em ambos os países, o compromisso político sustentado pode ser considerado um fator-chave para a criação de um ambiente favorável à integração da PAE, conforme apontado por diversos estudos (por exemplo, Torrens *et al*.)^(62)^.

### Brasil e Alemanha - sistemas incomparáveis? Por que contrastes geram aprendizados relevantes para a Prática Avançada de Enfermagem

Embora Brasil e Alemanha apresentem diferenças substanciais em termos demográficos, econômicos e estruturais dos sistemas de saúde (Quadro 1), o que limita comparações diretas dos caminhos de implementação da PAE, esse contraste é analiticamente valioso. A diversidade entre os contextos evidencia como diferentes arranjos institucionais, tradições profissionais e políticas moldam o desenvolvimento da PAE de formas distintas. Em vez de buscar simetria, a comparação permite ampliar a compreensão das condições sob as quais a PAE emerge (ou não) e das tradições que influenciam esse processo. Isso possibilita identificar estratégias sensíveis ao contexto e lições transferíveis que podem inspirar inovações em diferentes sistemas de APS.

### Limitações do estudo

Apesar de seu caráter informativo, este estudo apresenta limitações. Primeiramente, a PAE ainda está em processo de consolidação em ambos os países, de modo que a análise se baseia em conceitos em desenvolvimento, e não plenamente estabelecidos. Em segundo lugar, barreiras linguísticas também estiveram presentes, uma vez que alguns documentos relevantes, como aqueles relacionados à história da enfermagem ou à legislação nacional, estão disponíveis apenas em português ou alemão. No entanto, por meio da colaboração da equipe binacional, essa limitação foi superada. Por fim, não foram incluídos dados primários que captassem a perspectiva dos atores envolvidos, o que pode ter limitado a profundidade da análise. Entretanto, publicações relacionadas ao projeto mais amplo abordam essas perspectivas.

### Contribuições para a área da enfermagem

Esta análise comparativa do desenvolvimento da PAE na APS no Brasil e na Alemanha evidencia que a integração dessa prática depende de uma interação complexa entre fatores profissionais, educacionais, institucionais e políticos. Embora ambos os países tenham avançado na expansão do papel da enfermagem na APS, suas trajetórias refletem pontos de partida e condições sistêmicas distintas. No Brasil, a atuação consolidada dos enfermeiros na APS comunitária, aliada a uma infraestrutura acadêmica já estabelecida e em expansão, oferece uma base sólida para o desenvolvimento da PAE, apesar da ausência de regulamentação formal. Na Alemanha, reformas legislativas recentes e a existência de programas de formação em PAE indicam disposição para avançar, mas desafios estruturais persistem, especialmente relacionados à educação, à academização e à organização da APS.

Esse aspecto torna-se ainda mais relevante à medida que evidências demonstram a importância da PAE no cuidado direto ao paciente, incluindo melhorias nos desfechos assistenciais, especialmente no manejo de condições crônicas e no acesso aos serviços de saúde. Revisão sistemática^(61)^ mostra que a ampliação do escopo de prática da enfermagem está associada à redução do tempo de espera, maior capacidade de resolução na APS e resultados clínicos comparáveis aos dos médicos, além de altos níveis de satisfação dos pacientes. A PAE também contribui efetivamente para a adesão ao tratamento e a continuidade do cuidado^(62)^. Nesse sentido, as diferenças organizacionais e educacionais entre os contextos nacionais não apenas moldam a autonomia profissional, mas também influenciam diretamente a qualidade, eficiência e efetividade dos sistemas de saúde.

## CONSIDERAÇÕES FINAIS

A implementação da PAE não pode ser abordada de forma isolada. Sua implementação bem-sucedida depende da adoção de estratégias que alinhem reforma educacional, regulamentação profissional, organização dos sistemas de saúde e apoio político. Em vez de propor um modelo único, esta comparação reforça a importância de abordagens sensíveis ao contexto.

## Data Availability

Os dados de pesquisa encontram-se disponíveis no corpo do artigo.
